# Managing Agroecosystems for Soil Microbial Carbon Use Efficiency: Ecological Unknowns, Potential Outcomes, and a Path Forward

**DOI:** 10.3389/fmicb.2019.01146

**Published:** 2019-05-24

**Authors:** Cynthia M. Kallenbach, Matthew D. Wallenstein, Meagan E. Schipanksi, A. Stuart Grandy

**Affiliations:** ^1^ Department of Soil and Crop Sciences, Colorado State University, Fort Collins, CO, United States; ^2^ Natural Resource Sciences, McGill University, Sainte-Anne-de-Bellevue, QC, Canada; ^3^ Natural Resource Ecology Laboratory, Colorado State University, Fort Collins, CO, United States; ^4^ Department of Natural Resources and the Environment, University of New Hampshire, Durham, NH, United States

**Keywords:** soil ecology, carbon sequestration, CUE, agriculture, microbial biomass, crop diversity, tillage

## Abstract

Agricultural systems are increasingly managed for improving soil carbon (C) accumulation. However, there are limits to C returns in agricultural systems that constrain soil C accumulation capacity. Increasing the efficiency of how soil microbes process C is gaining interest as an important management strategy for increasing soil C and is a key feature of soil C dynamics in many new microbial-explicit models. A higher microbial C use efficiency (CUE) may increase C storage while reducing C system losses and is a fundamental trait affecting community assembly dynamics and nutrient cycling. However, the numerous ecological unknowns influencing CUE limit our ability to effectively manage CUE in agricultural soils for greater soil C storage. In this perspective, we consider three complex drivers of agroecosystem CUE that need to be resolved to develop effective C sequestration management practices in the future: (1) the environment as an individual trait moderator versus a filter, (2) microbial community competitive and faciliatory interactions, and (3) spatiotemporal dynamics through the soil profile and across the microbial lifecycle. We highlight ways that amendments, crop rotations, and tillage practices might affect microbial CUE conditions and the variable outcomes of these practices. We argue that to resolve some of the unknowns of CUE dynamics, we need to include more mechanistic, trait-based approaches that capitalize on advanced methods and innovative field research designs within an agroecosystem-specific context. By identifying the management-level determinants of CUE expression, we will be better positioned to optimize CUE to increase soil C storage in agricultural systems.

## Introduction

Annual agricultural ecosystems often deplete soil carbon (C) and release more reactive nitrogen (N) into the water and atmosphere than unmanaged, perennial ecosystems. Yet, we also rely on these ecosystems for global food security and they represent the largest stock of soil C we can directly manage to mitigate climate change. Can we resolve this dichotomy, creating a win-win scenario whereby agroecosystems remain productive while contributing to climate change mitigation? To address this grand challenge, agroecosystem soil biology is increasingly being managed to better regulate soil C and nutrient cycling (Wallenstein, 2017). Many approaches focus on soil C regeneration through increased residue returns and biomass production (cover crops) and decreasing C losses *via* reduced disturbance (no-till). The outcomes of these approaches do not always produce net C gains, and soil C accumulation is not always a linear function of inputs, in part because there are important and overlooked factors regulating the balance between inputs and outputs. One key determinant of this balance is the internal soil C cycling regulated by microbial C use efficiency (CUE), the proportion of C substrate microbes assimilate into new biosynthetic material relative to C lost out of the system as CO_2_. Microbial CUE directly affects the portion of C produced by net primary productivity (NPP) that becomes soil organic C, but we remain unable to predict its response to different combinations of agricultural practices.

Microbial CUE principles are emerging from laboratory evidence, conceptual and quantitative models, and, to some degree, field-based experiments (Cotrufo et al., 2013; Abramoff et al., 2018; Malik et al., 2018). Yet, the practicality of this knowledge for successfully implementing agricultural C sequestration requires addressing uncertainties in how CUE is manifested within an agroecosystem context. In this perspective, we highlight where our knowledge remains underdeveloped, lacking the intricacies of microbial community abiotic, biotic, and spatiotemporal interactions that might be central to accurately predicting management outcomes on CUE. We consider these uncertainties *vis-à-vis* potential management scenarios that may optimize CUE in agroecosystems. There are many methodological challenges and a lack of a commonly accepted CUE definition that have been recently addressed (e.g., Sinsabaugh et al., 2013; Geyer et al., 2016, 2019), but here we focus on the broader influences of land management on CUE that continue to challenge C sequestration management in agroecosystems. If these challenges can be resolved, we may be able to enhance CUE through agroecosystem management tools, and thus increase the efficiency of soil C processes.

## C Use Efficiency as a Core Trait to Achieving Sustainable Agriculture

Because harvesting limits the proportion of NPP-derived C that is returned to agricultural soils, increasing the efficiency of how microbes process C inputs is a critical approach for enhancing soil C storage. Recognizing this, many theoretical and process-based soil C models now represent CUE as a central regulator of soil C storage and decomposition dynamics (Cotrufo et al., 2013; Wieder et al., 2015; Abramoff et al., 2018). The influence of CUE on soil services is not just limited to soil C dynamics. Essentially a biosynthesis/uptake ratio, CUE determines microbial fitness and assembly (Shade et al., 2012; Wallenstein and Hall, 2012), crucial factors when considering agricultural practices intended to shift the soil microbiome (e.g., through microbial inoculums). Since CUE can be coupled to microbial cellular nutrient use efficiency (NUE), it may also influence soil N cycling as microbes adjust their CUE and NUE accordingly to maintain stoichiometric balances, affecting nutrient turnover and plant available N (Mooshammer et al., 2014).

Given the recognized importance of CUE, we present three themes we believe require deeper exploration to more effectively and predictably manage for optimal CUE in agroecosystems for increased soil C accumulation: (1) the environment as a trait moderator versus a filter, (2) biotic interactions, and (3) spatiotemporal dynamics.

## Examining The Complexities of C Use Efficiency in Agroecosystems

### The Environment As a Trait Moderator Versus Filter

**Figure 1:**
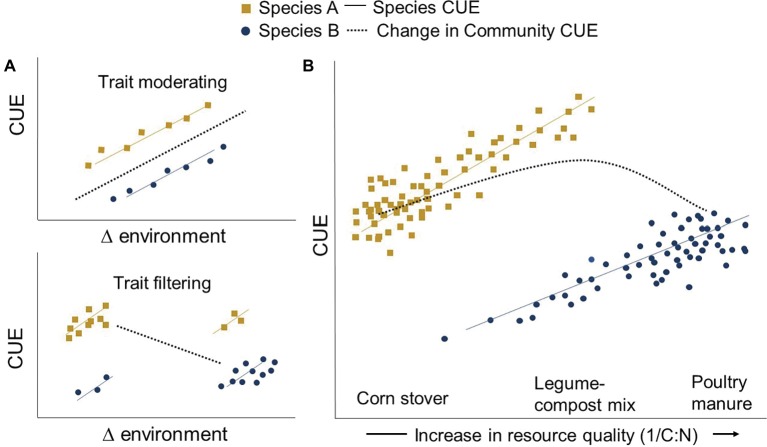
Trait moderating and trait filtering across a changing environment. Environmental change influences CUE by (1) modifying the existing community (trait modification) and/or (2) by selecting for a new community with different life histories (trait filtering). The community CUE response depends on whether trait moderating or filtering occurs [Panel **(A)**]. As an example, high-quality (1/C:N) inputs such as poultry manure may increase the intrinsic CUE of all community members (trait moderating) but may also shift the community toward one dominated by inefficient members (Species B) (trait filtering) [Panel **(B)**]. At the other end of the resource quality spectrum, applications of high C:N resources such as corn stover might shift the community toward more efficient microbes (Species A). Yet this community may still be C or N limited and thus have a lower CUE relative to its maximum potential. A community-level CUE “sweet spot” may exist in the middle that enhances the efficiency of all members without over-selecting for inefficient microbes or creating resource limitations.

Microbes are increasingly being grouped by their functional traits, any measurable heritable feature – including CUE – of an individual microbe, often affecting their fitness and performance (Krause et al., 2014). The interplay of the abiotic and biotic environment both filters and modifies how these traits emerge. Agricultural soil management (e.g., fertilization regime) can act as an environmental filter, determining the abundance of individual microbial taxa that collectively contribute to community-level CUE. For instance, long-term mineral fertilization can filter out some phyla such as *Acidobacteria* while selecting for a greater abundance of *Betaproteobacteria* (Ramirez et al., 2010; Francioli et al., 2016). But CUE is not determined by microbiome composition alone. Environmental changes can modify the existing community’s CUE through microbial trait plasticity. For example, stresses such as drought can increase maintenance energy requirements, thus lowering CUE (Schimel et al., 2007). As each individual approaches the edge of its trait window under changing conditions, competition from other taxa looms. The resultant community shift introduces new CUE limits to the system (Figure 1A). CUE is therefore an expression of microbial responses to the surrounding environment, and also the inherent physiological traits of the individuals of the community selected for by the environment (Allison, 2014).

One common way agricultural practices modify the soil environment is through altering microbial resource availability. Low C:N inputs like manure theoretically result in a higher organismal CUE (Sinsabaugh et al., 2013). Consequently, it has recently been proposed that agricultural practices providing high-quality inputs (e.g., low C:N) facilitate a higher CUE and, in turn, soil C accumulation *via* greater microbial biomass production (Cotrufo et al., 2013; Kallenbach et al., 2015; Wood and Bradford, 2018). However, several deviations to this principle have been observed, where higher C:N substrates or less bioavailable C corresponds to a relatively greater CUE (e.g., Lipson et al., 2009; Keiblinger et al., 2010; Creamer et al., 2015; Bölscher et al., 2016; Kallenbach et al., 2016; Bonner et al., 2018; Takriti et al., 2018). These observations could be explained by trait filtering, whereby a resource-poor environment selects for a greater abundance of efficient microbes, overriding the direct control of resource on individual CUE.

While field-based evidence remains thin (cf. Lipson, 2015), many classical microbiology culturing and chemostat experiments show wide variability in inherent microbial CUE limits (Geyer et al., 2016). These limits are often used to define life-history traits where, for example, microbes with a higher upper CUE limit are characterized as slower growing and dominate in resource-limited, oligotrophic environments (Roller and Schmidt, 2015). Populations thriving in more resource-rich environments are often described as having a relatively lower maximum CUE and sometimes higher cellular N requirements (Keiblinger et al., 2010). Thus, any shift in community composition toward or away from populations with intrinsically different CUE could alter how community-level CUE is manifested (Figure 1A). Scenarios where resource inputs promote trait filtering may thus explain the observed variation in CUE response to resource quality. High-quality resource inputs may improve individual CUE (trait modification) but could also over-select for populations with inherently lower CUE limits, lowering community-level CUE (Figure 1B). This potential shift between resources promoting individual versus community CUE needs to be considered when interpreting new conceptual and quantitative models that link resource inputs to CUE and C accumulation (e.g., Cotrufo et al., 2013; Campbell et al., 2016). The challenge in targeting an optimal community-level CUE by altering resource availability is to better understand the threshold where the environment shifts from acting as an individual trait moderator to a community filter.

A diversity of inputs representing a wide range of C and nutrient availability and chemistry might facilitate a balance between individual and community-level CUE optimization (Figure 1B). Practices such as diversifying crop rotations or mixing legume cover crop biomass with corn or wheat residues could provide resources that promote species with different life histories to coexist. Each member can thereby approach its individual maximum CUE potential (Figure 1B). Thus, community CUE might be maximized just before a threshold in community shift occurs, where a diversity of inputs provides resources for each member to realize their optimum CUE without shifting toward an overabundance of inefficient microbes.

### Biotic Interactions

#### Microbial Competition

Biotic interactions directly alter how and when fitness traits are expressed. It is thus reasonable to assume that such interactions will modulate individual and community CUE (Frey et al., 2001; Buchkowski et al., 2017). In soils, many different competition relationships with varying strength likely occur over space and time, yet the consequences for CUE are relatively unexplored. In one of the few studies to directly test this, fungal competition increased CUE but only under indirect competition, when no single species was universally weak, while direct competition decreased CUE (Maynard et al., 2017). The greater the system heterogeneity, the more likely indirect competition will occur (Allesina and Levine, 2011). If CUE is positively related to indirect competition, as opposed to direct competition favored in more homogenous environments, greater soil heterogeneity may foster a higher CUE. More structured heterogenous environments also theoretically favor *k*-strategists outcompeting *R*-strategists, characterized by a relatively lower CUE (Pfeiffer et al., 2001). The CUE response to competition could thus depend on which competitive behavior dominates in the soil and who wins. But are agroecosystems tractable enough to feasibly manage the nuances of microbial competition for distinct CUE outcomes?

As soils are already heterogenous systems, efforts to manipulate competition dynamics for optimal CUE might be most effective in agroecosystems that are relatively limited in their inherent heterogeneity (e.g., young soil, 1:1 minerals, high sand content). In such cases, the above competitive dynamics expected to favor a higher CUE might not be well supported. Practices such as tillage reduction could create a more heterogenous environment with potentially positive effects on CUE *via* improved aggregation, increased pore size diversity, and by altering distribution of “hot spots.” Further, leaving more residues on the soil surface creates distinct resource zones through the soil profile (Williams et al., 2016). Chemical diversity also enhances soil heterogeneity (Nunan, 2017). Diversification of inputs or inputs with a diversity of unique chemical compounds may similarly promote indirect over direct competition or favor *k*-strategist decomposers, with potentially positive outcomes for CUE.

#### Microbial Facilitation

Microbes can enhance one another’s fitness through faciliatory interactions such as cross-feeding – for example, when one population alters a resource making it more bioavailable for another population (McIntire and Fajardo, 2014). Microbial facilitations can increase the spatial extent of a realized niche and generate new metabolic niches, valuable where resources would otherwise be limiting (Bruno et al., 2003). For example, at later decomposition stages, most bioavailable residue C is exhausted but accumulated microbial biomass may allow late-stage decomposers to maintain a relatively high CUE if that labile microbial biomass turns over (Kaiser et al., 2014). Assumptions that CUE declines with decomposition time might not necessarily be accurate when considering the range of faciliatory interactions that occur during community succession and the by-products left behind by preceding communities.

For facilitation to occur, microbes also need to occupy a similar space to access and benefit from newly generated resources, requiring close microbial interactions (Folse and Allison, 2012). More interactive microbial networks are thought to improve ecosystem function and potentially CUE (de Vries and Wallenstein, 2017; Morriën et al., 2017). In much of the soil though, microbial abundances are low, access to resources are limited, and metabolism is highly constrained. Practices that increase the amount and diversity of inputs into the resource-limited bulk soil may enhance both microbial abundances and niches that facilitate closer networks and cooperation. For example, instead of surface compost applications, incorporating compost into the soil could promote a microbial habitat analogous to the rhizosphere where more faciliatory interactions are likely to occur (Wallenstein, 2017).

Microbial connectivity can also be influenced through other management approaches. The rhizosphere area can be expanded through diverse cropping systems with distinct rooting depths and architecture that minimize the bulk soil space where faciliatory interactions may be limited and CUE depressed. Integrated livestock systems can also affect the spatial and temporal distribution of root C, though grazing effects on root dynamics vary considerably with grazing management system (Piñeiro et al., 2009). Enhancing the movement of dissolved organic C and water into the bulk soil, by altering wetting (*via* irrigation), redox, and rooting patterns could further stimulate greater microbial connectivity and cooperation by increasing resource availability and microbial movement in water films (Manzoni et al., 2014).

Microbial community interactions are varied, and we do not yet know which types of interactions (e.g., competition versus facilitation) might support higher community CUE, let alone how to target desired biotic interactions through agricultural practices. In reality, biotic interactions might be sparse given that microbes occupy a small portion of the total soil surface area and have limited movement (Nunan, 2017). In the bulk soil, environmental controls may be the dominant influence on CUE, with biotic controls stronger in the rhizosphere or where connectivity is greater (Sokol et al., 2019). The needs for evaluating biotic effects on CUE are first to determine where and when biotic controls override abiotic factors, which and when a specific biotic interaction increases CUE and over what time period, and ultimately if we can effectively facilitate these biotic interactions through management.

### Spatiotemporal Dynamics

#### C Use Efficiency Through the Soil Profile

Isolating management efforts to the crop rooting zone may result in soil C trade-offs at deeper soil depths, affecting total soil C sequestration. If we target management practices that enhance CUE at the soil surface, we may inadvertently reduce CUE at deeper depths. The environment becomes increasingly harsh for microbes with increasing soil profile depth that likely favors more efficient oligotrophs. However, even with an intrinsically higher CUE, oligotrophs might not be functioning at their optimum CUE, or may even be dormant, given high resource constraints deeper in the soil.

Reducing tillage and maintaining crop residues on the soil surface might intensify these conditions, further constraining the oligotrophic population’s CUE. Rather, increasing organic materials deeper in the soil profile with tillage could enhance the fitness and CUE of the microbial community at depth. Indeed, van Groenigen et al. (2013) have shown declines in CUE with depth under reduced tillage wheat systems but CUE increases with depth under conventional tillage. Introducing perennials into cropping systems or managing livestock grazing to facilitate greater rooting depths will also increase organic inputs at depth. In a pasture-based system, Spohn et al. (2016) show no effect of depth on CUE, perhaps due to a large belowground investment and subsequently reduced oligotrophic zone. While there are multiple benefits to reducing tillage, some data suggest that reduced tillage does not consistently increase total C stock but rather results in more C concentrated near the soil surface (Powlson et al., 2014). Perhaps declines in CUE with depth are greater under no-till systems and explain why we do not always observe overall increases in soil C. We do not argue for increasing tillage but believe that understanding tillage effects on the final fate of crop residues may involve underappreciated, complex microbial mechanisms and that tillage may have benefits to improving CUE at greater soil depths.

Still, optimizing CUE through more of the soil profile may have its trade-offs. For instance, priming of existing soil organic matter at depth may occur in tandem with increasing CUE (Fontaine et al., 2007). Currently, there is limited information describing CUE with depth and thus several outcomes can be considered. For policy makers, climate change forecasters, and soil extension agents, this is an unproductive position to be in. To help define systems that increase soil C stocks and do not simply redistribute it, we need to invest more efforts into understanding management effects on CUE beyond the crop rooting zone and their potential trade-offs.

#### C Use Efficiency Temporal Variability

The fate of C assimilated into microbial biomass depends on the soil stabilization capacity (e.g., texture and mineralogy), but also changes over time, as CUE likely fluctuates with temporal environmental changes. The C used for biosynthetic microbial materials may not necessarily remain within a cell, as it can be later exploited for catabolic processes during times of resource limitations (Joergensen and Wichern, 2018). Agroecosystems oscillate between periods of high and low resource availability and microbes are most often C limited, especially during the nongrowing seasons. As such, soil microbes spend much of their time in a state of dormancy, slow growth, or in feast-to-famine cycles. Though energy is conserved during dormancy, significant energetic costs occur during transition into and out of dormancy (Lennon and Jones, 2011). During dormancy, biomass production halts and C previously stored in biosynthesized materials can be recycled or spent during maintenance metabolism (Kempes et al., 2017). Accordingly, with shorter dormancy periods, relatively more new biomass can be produced and reductions in endogenous metabolism may occur. Moreover, dormancy may delay microbial biomass turnover, reducing the production rate of dead microbial cells. This, however, represents another area of uncertainty. While it is reasonable to predict that dead microbial cells are more likely to stabilize on minerals than living cells, more research is needed to understand the relationships between CUE and microbial biomass turnover that can significantly influence both the short- and long-term rates of microbial C accumulation (Hagerty et al., 2014).

**Figure 2:**
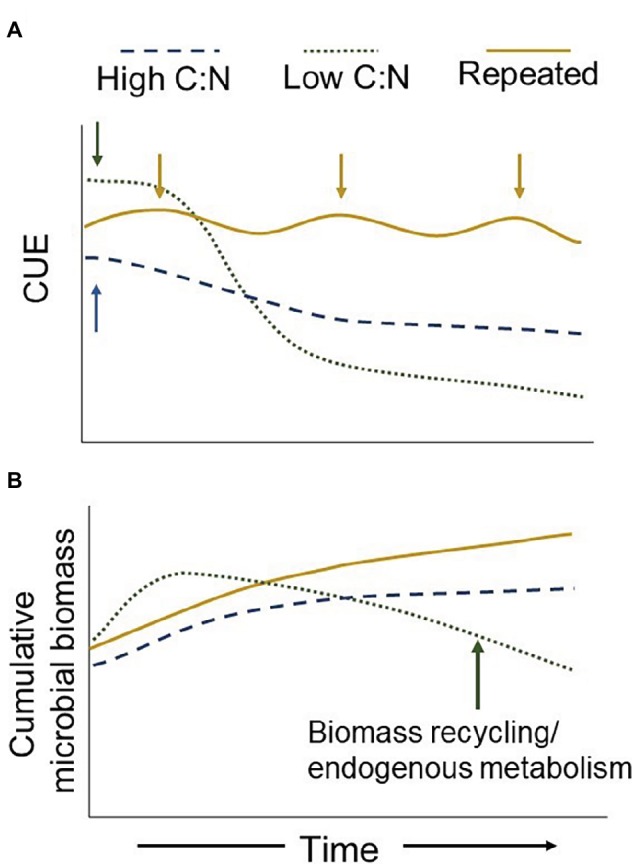
Theoretical temporal dynamics of CUE under different resource inputs and timing. Single additions (arrows) of high-quality (low C:N) inputs early in the season result in a higher initial CUE but faster decomposition rate [Panel **(A)**]. Without replenishment, the microbial community will experience C limitations more quickly, reducing CUE, biomass production, and increasing biomass recycling [Panels **(A,B)**]. Such a decline in off-season microbial biomass production counteracts the benefits of a relatively higher early-season CUE associated with higher quality residues. This potentially reduces the overall annual amount of C inputs ending up as microbial stable C. Alternatively, a lower quality input results in a lower initial CUE but continues to provide a C source longer due to slower decomposition. Thus, annual cumulative CUE and biomass production may be relatively greater with lower quality inputs [Panels **(A,B**)]. Perennial or cover crop systems (repeated inputs) provide a more constant resource, effectively alleviating C limitations throughout the year, allowing microbes to delay metabolic dormancy and maintain a relatively higher CUE.

Including cover crops, managing compost inputs as multiple split applications, or integrating perennial crops into rotations could moderate off-season C limitations, reducing the time microbes are dormant (Figure 2). Steep declines in CUE associated with resource exhaustion may also be reduced by extending the length of time crop residues and other organic inputs persist in the soil (Figure 2). Maintaining a relatively lower CUE but a longer period of metabolic activity, may result in more microbial-derived C accumulation than managing for a higher CUE for a shorter time (Figure 2). A more active community for a longer time period evokes the soil C dilemma – “should we hoard it or use it” (Janzen, 2006) – and reminds us that many agroecosystems are ultimately constrained by low C inputs relative to their natural counterparts. However, here, under higher C:N inputs, the microbial community is active longer but presumably growing slower (Figure 2). Consequently, the same quantity of added C may still last longer compared to a community that becomes dormant earlier after rapidly metabolizing the same quantity of added C (Figure 2). Moreover, a higher CUE does not necessarily translate to more C transferred to microbial biomass. A relative increase in CUE can occur with lower gross C uptake coinciding with lower respiration rates but no change to biomass production. In considering soil C accumulation *via* microbial inputs, such a scenario would have no meaningful benefit. Thus, it is important to not only understand CUE in terms of the amount of C being processed but also the rate, and finally the fate of assimilated C over time.

## Moving Forward

### Basic Science and Trait-Based Research for Agroecosystems

Microbial CUE has system-wide effects on agricultural sustainability, yet how CUE is manifested within an agroecosystem context is idiosyncratic given the numerous interactions that arise between the environment, the individual microbe, microbial community membership, and management changes. We can begin to unravel the complexity and challenges of predicting management controls on CUE by directing efforts toward more basic research in soil agroecosystems. Agricultural experiments designed to address more fundamental ecological questions could isolate leverage points to manipulate for desired agroecosystem services (Fierer et al., 2009). Moreover, such experiments would identify trends and principles useful for predicting system responses (e.g., CUE) to disturbances such as tillage and drought.

One path to advance fundamental ecological concepts in agroecosystems is to apply a trait-based approach. Already recommended in multiple microbial models (Allison, 2012; Wieder et al., 2015; Treseder et al., 2018), a trait-based framework has had slow adoption in agricultural ecosystems which arguably stand to benefit the most given the high degree of human-induced variability and malleability for ecosystem services. Understanding the underpinnings and dynamics of microbial traits could be an effective way forward in shaping our principles of agroecosystem soil C and N cycling (Krause et al., 2014). More so than community composition and perhaps even function, microbial traits such as stress tolerance, growth rate, dormancy, and CUE can improve predictions of microbial performance in response to management shifts and affect outcomes of community assembly and competition (crucial for pest management and microbial inoculant applications) (Wallenstein and Hall, 2012; Fontana et al., 2018). A framework focused on linkages between management and microbial traits will allow us to better describe, predict, and manage the relationships among critical soil services, the microbes that drive them, and the environment under which they are manifested.

### Innovation and Creativity

Advanced analytical approaches such as network analysis, stable isotope probing (SIP), flow cytometry, and nanoSIMS are already being applied to better link biogeochemistry and the environment with microbial traits, spatial organization, and ecology (Stiehl-Braun et al., 2011; Behrens et al., 2012; Starr et al., 2018). Recent studies using DNA-SIPs and “omics” approaches have, for example, linked plant exudation and microbial C uptake traits to microbial community assembly dynamics (Zhalnina et al., 2018) and demonstrated bacterial faciliatory interactions important for taxon-specific soil C transformations (Pepe-Ranney et al., 2016). Increasing the intellectual investment and training in these methods, especially within an agricultural context, is an obvious requirement moving forward. Less obvious is the need for these techniques in combination with creative field experiments intended to test mechanistic hypotheses, in addition to purely monitoring management outcomes (Fierer et al., 2009). For example, soil transplant experiments have provided valuable insight on microbial stability and decomposition dynamics (Waldrop and Firestone, 2006; Liang et al., 2015), but are rare and could help inform the boundary between abiotic and biotic controls on CUE. Similarly, we need to emphasize capturing spatial and temporal resolution to gain a more systems-based perspective of how microbes are interacting with each other and the environment (Gonzalez et al., 2012; Schipanski et al., 2014).

Given that principles (and methods) around CUE remain undeveloped and poorly tested, directing soil management outcomes based on CUE assumptions should be done with caution. The reality is that the current state of knowledge is too limited to accurately predict CUE responses in the field. As we move forward, recommendations to improve soil C sequestration and effectively engineer rhizosphere microbiota and enhance nutrient efficiency will require increased efforts in understanding fundamental soil-microbial processes within an agroecosystem context, emphasize a trait-based approach, and increase the use of advanced methods and innovative field research designs. By resolving CUE unknowns associated with abiotic and biotic forces and temporal and spatial dynamics, we will be better positioned to predict management outcomes for CUE and thus more reliable practices to increase soil C storage.

## Author Contributions

CK, MW, MS, and AG contributed to ideas and manuscript writing and editing.

### Conflict of Interest Statement

The authors declare that the research was conducted in the absence of any commercial or financial relationships that could be construed as a potential conflict of interest.
